# Longitudinal changes in the abundance of IgA1 *O-* and *N*-glycoforms in IgA nephropathy

**DOI:** 10.1007/s10157-025-02659-y

**Published:** 2025-04-07

**Authors:** Masaya Hirayama, Yukako Ohyama, Yudai Tsuji, Tetsuro Enomoto, Midori Hasegawa, Naotake Tsuboi, Jan Novak, Kazuo Takahashi

**Affiliations:** 1 Department of Biomedical Molecular Sciences, Fujita Health University School of Medicine, 1-98 Dengakugakubo, Kutsukake-Cho, Toyoake, Aichi 470-1192 Japan; 2 Department of Pathology and Cytopathology, Fujita Health University School of Medical Sciences, 1-98 Dengakugakubo, Kutsukake-Cho, Toyoake, Aichi 470-1192 Japan; 3 Department of Nephrology, Fujita Health University School of Medicine, 1-98 Dengakugakubo, Kutsukake-Cho, Toyoake, Aichi 470-1192 Japan; 4 Oriental Yeast Co., Ltd, 50 Kanou-Cho, Nagahama, Shiga 526-0804 Japan; 5 Department of Microbiology, University of Alabama at Birmingham, 1720 2nd Ave South, Birmingham, AL 35294 USA

**Keywords:** IgA nephropathy, IgA1, *O*-Glycan, *N*-Glycan, Mass spectrometry

## Abstract

**Background:**

IgA nephropathy (IgAN) is the most common type of primary glomerulonephritis. Elevation in the blood levels of aberrantly glycosylated IgA1 is a crucial initial step in IgAN pathogenesis. Here, we aimed to determine the longitudinal changes in the serum levels of IgA1 *O*- and *N*-glycoforms in patients with IgAN receiving different treatments.

**Methods:**

We enrolled Japanese patients diagnosed with primary IgAN: 10 patients who underwent tonsillectomy and corticosteroid therapy (T-CST), 7 who received corticosteroid therapy (CST), 8 who received conservative therapy (CO), and 5 with other renal diseases who received corticosteroid therapy (ORD) as disease controls. IgA was purified from patient sera collected at diagnosis and post-treatment. After sample preparation, *O*-glycoforms of the hinge region (HR) and *N*-glycoforms of the fragment crystallizable region were analyzed using high-resolution mass spectrometry (MS).

**Results:**

The MS analysis of *O*-glycoforms of IgA1 showed that the relative abundance of IgA1 with 3GalNAc3Gal, which we previously identified as a characteristic IgA1 *O*-glycoform in IgAN, decreased post-treatment only in the T-CST group (*P* = 0.0195). Regarding *N*-glycoforms, the relative abundance of fucosylated *N*-glycan at asparagine (Asn)^340^ increased in the IgAN group compared with that in the ORD group (*P* = 0.0189) and decreased post-treatment only in the T-CST group (*P* = 0.0195).

**Conclusion:**

The MS analysis of *O*- and *N*-glycoforms of IgA1 revealed substantial changes in their abundance in the T-CST group but not in the CST, CO, and ORD groups. Our study provides new insights into how specific treatments alter the IgA1 glycoform abundance.

**Supplementary Information:**

The online version contains supplementary material available at 10.1007/s10157-025-02659-y.

## Introduction

IgA nephropathy (IgAN) is the most frequent primary glomerulonephritis worldwide and is characterized by the glomerular mesangial co-deposition of IgA1, IgG, and complement C3 [1]. The clinical and pathological disease characteristics of IgAN are heterogeneous, with 20–40% of patients progressing to kidney failure within 20 years of diagnosis [2]. In 2011, a multi-hit mechanism was proposed for the pathogenesis of IgAN [3]. This hypothesis postulates that patients with IgAN have elevated blood levels of aberrantly glycosylated IgA1, the main autoantigen in the pathogenesis of IgAN.

**Fig. 1:**
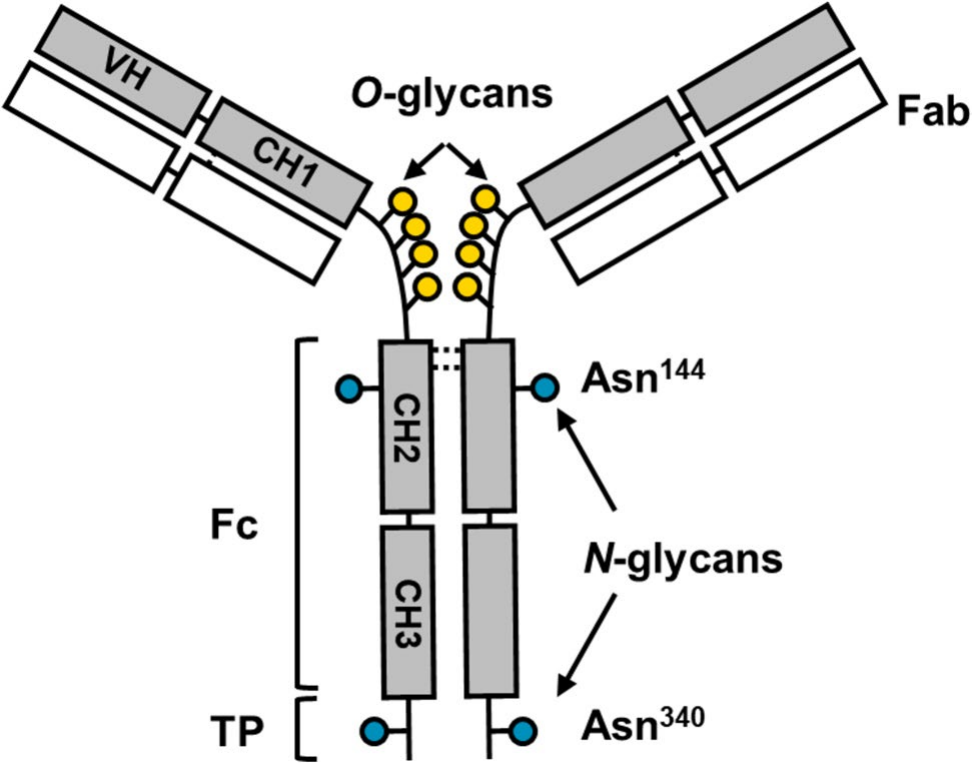
Molecular structure of IgA1 and its glycosylation sites. IgA1 has an extended hinge region (HR) with nine potential *O*-glycosylation sites (serine or threonine). Of these 9 potential sites, 3–6 are usually occupied. IgA1 has two *N*-glycosylation sites at asparagine (Asn)^144^ and Asn^340^. *VH* variable heavy chain, *CH1* constant heavy chain 1, *CH2* constant heavy chain 2, *CH3* constant heavy chain 3, *TP* tailpiece, *Fc* fragment crystallizable

Human IgA1 has two *N*-glycosylation sites in the constant heavy chain 2 (CH2) region and in the tailpiece (TP), at asparagine (Asn)^144^ and Asn^340^ [4–7]. Furthermore, IgA1 has 3–6 *O*-glycans in its hinge region (HR), similar to other hominid primates (Fig. 1) [8]. As the predominant mesangial IgA in IgAN is IgA1, characterization of the features of IgA1 glycosylation in patients with IgAN has been performed using lectin enzyme-linked immunosorbent assay (ELISA) [9]. Reduced terminal galactosylation of the IgA1 HR *O*-glycan has been demonstrated in IgAN based on the results of lectin ELISA using *Helix aspersa* agglutinin (HAA; a lectin that specifically recognizes the terminal *N*-acetylgalactosamine (GalNAc) of *O*-glycans) [9]. However, no abnormalities have been observed in *N*-linked glycosylation of IgA1 in IgAN, based on the results of lectin ELISA using *Triticum vulgaris* agglutinin (which binds the terminal *N*-acetylglucosamine (GlcNAc) of *N*-glycans) and *Erythrina crystagalli* agglutinin (which binds the terminal D-galactose (Gal) of *N*-glycans) [9]. These results were validated in a large number of cases using HAA, and it was confirmed that the galactose-deficient IgA1 level is elevated in the sera of most patients with IgAN [10, 11].

Recently, the molecular features of IgA1 *O*- and *N*-glycans in patients with IgAN have been reported using high-resolution mass spectrometry (HRMS) [12–15]. Dotz et al. reported that changes in galactosylation, sialylation, bisection, fucosylation, biantennary forms of *N*-glycans, and sialylation of *O*-glycans are associated with IgA1 in IgAN patients [12]. Galactose deficiency in IgA1 HR *O*-glycans was not identified as a feature of IgAN in that study. Asian research groups have previously reported that elevated levels of IgA1 with 3GalNAc3Gal and IgA1 with reduced number of *O*-glycans to be characteristic of IgA1 in patients with IgAN, and this feature is associated with pathological changes in IgAN [13–15]. Therefore, *O*- and *N*-glycosylation of IgA1 may be potential biomarkers for IgAN. However, it is still unknown whether the *O*- and* N*-glycosylation of IgA1 changes longitudinally according to the treatment protocol.

In the clinical setting, patients with IgAN present a wide range of clinical manifestations, ranging from asymptomatic microscopic hematuria to rapidly progressive glomerulonephritis. For the successful management of IgAN, it is important to identify patients with poor prognosis and provide appropriate treatment [16]. Hence, treatment options such as optimized supportive care including renin–angiotensin system (RAS) blockade or/and glucocorticoid therapy for 6 months are preferred based on the clinical and pathological findings [16, 17].

To determine possible longitudinal changes in the *O*- and *N*-glycosylation of IgA1 based on treatment protocols, we compared the differences in the abundance of IgA1 glycoforms between pre- and post-treatment across different treatment groups.

## Materials and methods

### Study population

**Fig. 2:**
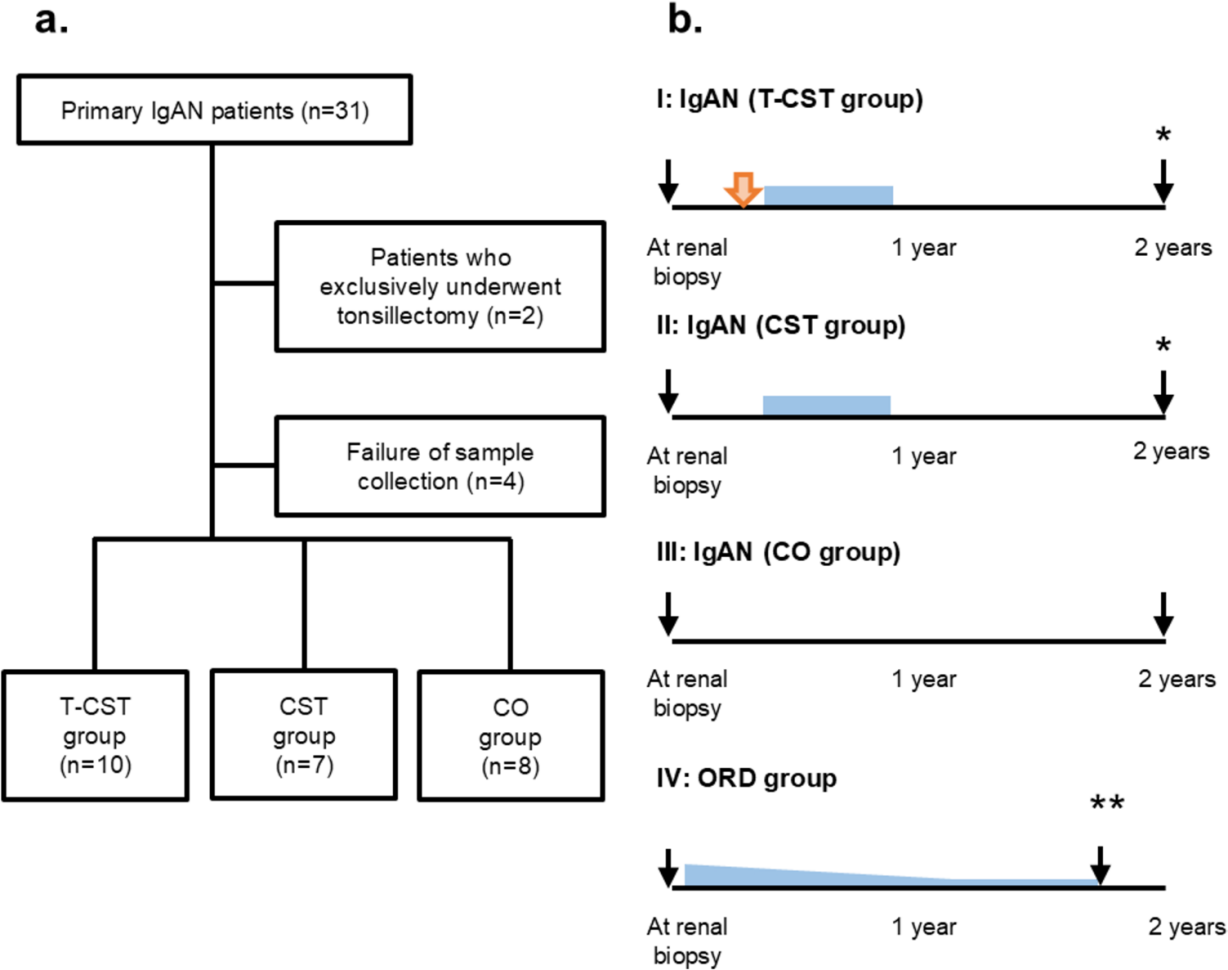
Study design. **a** Diagram of the study population. Patients with primary IgA nephropathy (IgAN) diagnosed based on an evaluation of renal biopsy were enrolled. Treatment options were selected as shown in the flowchart in Supplementary Fig. S1. After excluding patients who received exclusive tonsillectomy treatment, 10 patients were enrolled in the T-CST group, 7 in the CST group, and 8 in the CO group. *IgAN* IgA nephropathy, *T-CST* tonsillectomy and corticosteroid therapy, *CST* corticosteroid therapy, *CO* conservative therapy. **b** Timing of sample collection. The timing of sample collection is indicated with black arrows. The timing of palatine tonsillectomy is indicated with the orange arrow and the duration of corticosteroid administration is indicated with the pale-blue box. Baseline serum samples were collected during renal biopsy. Post-treatment sera were collected 2 years after the diagnosis for patients in the T-CST, CST, and CO groups. If treatment onset was delayed, samples were collected 1 year after the cessation of immunosuppressive therapy in the T-CST and CST groups (*). In the ORD group, serum samples were collected more than 1 year after diagnosis at the last follow-up. The patients were in remission and received oral corticosteroids at less than 5 mg/day (**). *ORD* other renal diseases

Thirty-one Japanese patients with biopsy-confirmed primary IgAN between 2017 and 2019 at Fujita Health University Hospital (FHU) were screened. Treatment options were selected based on the treatment selection flowchart of the FHU (Supplementary Fig. S1). We enrolled 10 patients who underwent both tonsillectomy and corticosteroid therapy (T-CST group), 7 who were exclusively treated with corticosteroid (CST group), and 8 who were on conservative therapy (CO group) (Fig. 2a). Five patients with other renal diseases (two with membranous nephropathy and three with minimal change nephrotic syndrome) who received corticosteroid therapy (ORD group) were enrolled as disease controls.

In the T-CST and CST groups, corticosteroid therapy was administered based on the Pozzi protocol modified for Japanese patients [18, 19]. Patients assigned to the CO group received only supportive therapy, which included RAS inhibitor administration. Patients in the ORD group were treated with only oral corticosteroids, except for one patient who was treated with corticosteroids and cyclosporine. Serum samples collected around the time of renal biopsy were used as baseline samples. The treatment course and timing of sample collection are shown in Fig. 2b.

### Purification of IgA1 and sample preparation for MS

Purification of IgA1 and sample preparation for MS were performed as previously described [5, 13].

### Liquid chromatography–mass spectrometry analysis for profiling IgA1 HR *O*-glycoforms and Fc *N*-glycoforms

Desialylated and trypsin-digested IgA (500 ng) was analyzed using on-line liquid chromatography (LC)–MS as previously described [13].

### Analysis of IgA1 *O*- and *N-*glycoform profiling data

All spectra were analyzed using Xcalibur Qual Browser 2.2 (Thermo Fisher Scientific). The RA of each *O*-glycopeptide and the mean number of GalNAc (or Gal) residues per HR were determined using previously reported formulae [5, 13]. Identification of IgA1 *N-*glycopeptides at Asn^144^ and Asn^340^ was performed manually, based on the mass values of the trypsin-digested IgA1 fragment crystallizable (Fc) region amino acid sequences, using the GlycoMod tool (http://www.expasy.org). The RA of each *N*-glycoform were determined by the area under the curve (AUC) of each glycopeptide-extracted ion chromatogram (XIC).

### Enzyme-linked immunosorbent assay

Total serum IgA level was measured using an enzyme-linked immunosorbent assay (ELISA) kit (88–50,600; Thermo Fisher Scientific, Waltham, MA, USA) according to the manufacturer’s instructions.

### Quantification and statistical analysis

Statistical analyses and graphing were performed using GraphPad Prism 9 and RStudio software (version 3.6.3). Pre- and post-treatment data were compared using a paired *t* test or Wilcoxon matched-pairs signed-rank test, depending on whether the variables were distributed normally. For multiple comparisons, a one-way analysis of variance (ANOVA) was performed depending on the normal distribution of the variables. Categorical variables are expressed as percentage and compared using Fisher’s exact test. Statistical significance was set at *P* < 0.05.

## Results

### Clinical and laboratory information

The demographic and clinical characteristics of the total cohort, consisting of the T-CST, CST, CO, and ORD groups, are shown in Table 1. The urinary protein levels and red blood cell counts decreased significantly post-treatment in the T-CST group (*P* = 0.0020 and *P* < 0.0001, respectively), but not in the CST (*P* > 0.9999,* P* = 0.1923) and CO groups (*P* = 0.0567, *P* = 0.3665). All patients in the ORD group achieved remission post-treatment. Regarding histological findings, the crescent scores C1 and C2 were more frequent in the T-CST and CST groups than in the CO group (*P* = 0.0029 and *P* = 0.0014, respectively), but there were no significant differences between the T-CST and CST groups (*P* > 0.9999). More patients in the T-CST group exhibited endocapillary hypercellularity (E1 score) than those in the CO group (*P* = 0.0040), although there was no significant difference between the T-CST and CST groups (*P* = 0.1534). In contrast, mesangial hypercellularity (M1), segmental glomerulosclerosis (S1), and tubular atrophy/interstitial fibrosis (T1,2) scores were similar among the groups (Table 1).

**Table 1:** Clinical characteristics of patients pre- and post-treatment in the four groups

	T-CST group (RBX)(*n* = 10)	T-CST group (2years) (*n* = 10)	*P* value	CST group (RBX) (*n* = 7)	CST group (2years) (*n* = 7)	*P value*	CO group (RBX) (*n* = 8)	CO group (2years) (*n* = 8)	*P value*	ORD group (RBX) (*n* = 5)	ORD group (CR) (*n* = 5)	*P value*	Comparison at diagnosis in T-CST, CST, CO, (ORD) groups * P* value
Age, years (quartile range)	35 (24.3–47.3)	37.5 (27.3–50.0)	< 0.0001^****^	39 (27.0–59.5)	42 (29.0–62.0)	< 0.0001^****^	46.5 (34.3–62.5)	49 (36.5–64.5)	< 0.0001****	50 (47.0–56.0)	51 (48.5–59.5)	0.0217*	0.2754
Men (%)	5 (50.0)	N/A	N/A	4 (57.1)	N/A	N/A	1 (12.5)	N/A	N/A	1 (20.0)	N/A	N/A	0.2002
MAP, mmHg (quartile range)	96.0 (87.9–102.9)	93.7 (79.9–99.1)	0.4119	94 (87.8–102.7)	85.3 (79.3–103.2)	0.0625	93.2 (83.1–101.8)	86.0 (76.4–95.9)	0.0920	95.0 (90.0–96.7)	93.7 (86.7–106.0)	0.8237	0.9116
Cr, mg/dL (quartile range)	0.98 (0.77–1.34)	0.95 (0.73–1.35)	0.9144	0.88 (0.68–1.11)	0.74 (0.67–1.07)	0.8348	0.92 (0.81–1.28)	0.89 (0.84–1.20)	0.7734	0.77 (0.65–0.84)	0.65 (0.63–0.68)	0.3725	0.2059
eGFR, mL/min/1.73 m^2^ (quartile range)	66.7 (44.3–85.2)	71.4 (43.3–81.8)	0.5677	58.6 (43.3–81.8)	68.8 (56.3–93.7)	0.4521	55 (44.1–68.9)	54.9 (43.4–64.8)	0.5505	73.9 (59.2–74.7)	76.9 (70.7–82.1)	0.6067	0.5051
UP, g/gCr (quartile range)	1.35 (0.26–1.88)	0.08 (0.02–0.21)	0.0020**	0.53 (0.50–1.00)	0.69 (0.33–0.90)	> 0.9999	1.12 (0.50–1.73)	0.39 (0.20–1.13)	0.0567	7.8 (6.25–8.34)	0.051 (0.00–0.06)	0.0016**	< 0.0056**
Urinary red blood cell count, more than 10/HPF, yes (%)	9 (90.0)	0 0.0	< 0.0001***	7 (100)	4 (57.1)	0.1923	5 (62.5)	2 (25.0)	0.3665	1 (20.0)	0 (0.0)	> 0.9999	0.0101*
M1 score (%)	6 (60.0)	N/A	N/A	2 (28.6)	N/A	N/A	7 (87.5)	N/A	N/A	N/A	N/A	N/A	0.0671
E1 score (%)	7 (70.0)	N/A	N/A	2 (28.6)	N/A	N/A	0 (0.0)	N/A	N/A	N/A	N/A	N/A	0.0079**
S1 score (%)	8 (80.0)	N/A	N/A	4 (57.1)	N/A	N/A	6 (75.0)	N/A	N/A	N/A	N/A	N/A	0.5713
T (1,2) score (%)	4 (40.0)	N/A	N/A	1 (14.3)	N/A	N/A	2 (25.0)	N/A	N/A	N/A	N/A	N/A	0.4257
C (1,2) score (%)	9 (90.0)	N/A	N/A	7 (100)	N/A	N/A	1 (12.5)	N/A	N/A	N/A	N/A	N/A	0.0002***

Data are presented as median (interquartile range) or number (%). *RBX* renal biopsy, *T-CST* tonsillectomy and corticosteroid therapy, *CST* corticosteroid therapy, *CO* conservative therapy, *ORD* other renal diseases, *MAP* mean arterial pressure, *Cr* serum creatinine concentration, *eGFR* estimated glomerular filtration rate, *UP* urinary protein-to-creatinine ratio, *HPF* high power field, *M* mesangial proliferation, *E* endocapillary proliferation, *S* segmental glomerulosclerosis, *T* interstitial fibrosis/tubular atrophy, *C* crescents, * 0.01 ≤ *P* < 0.05, ** 0.001 ≤ *P* < 0.01, *** 0.0001 ≤ *P* < 0.001, **** *P* < 0.0001

### Changes in serum IgA levels pre- and post-treatment

**Fig. 3:**
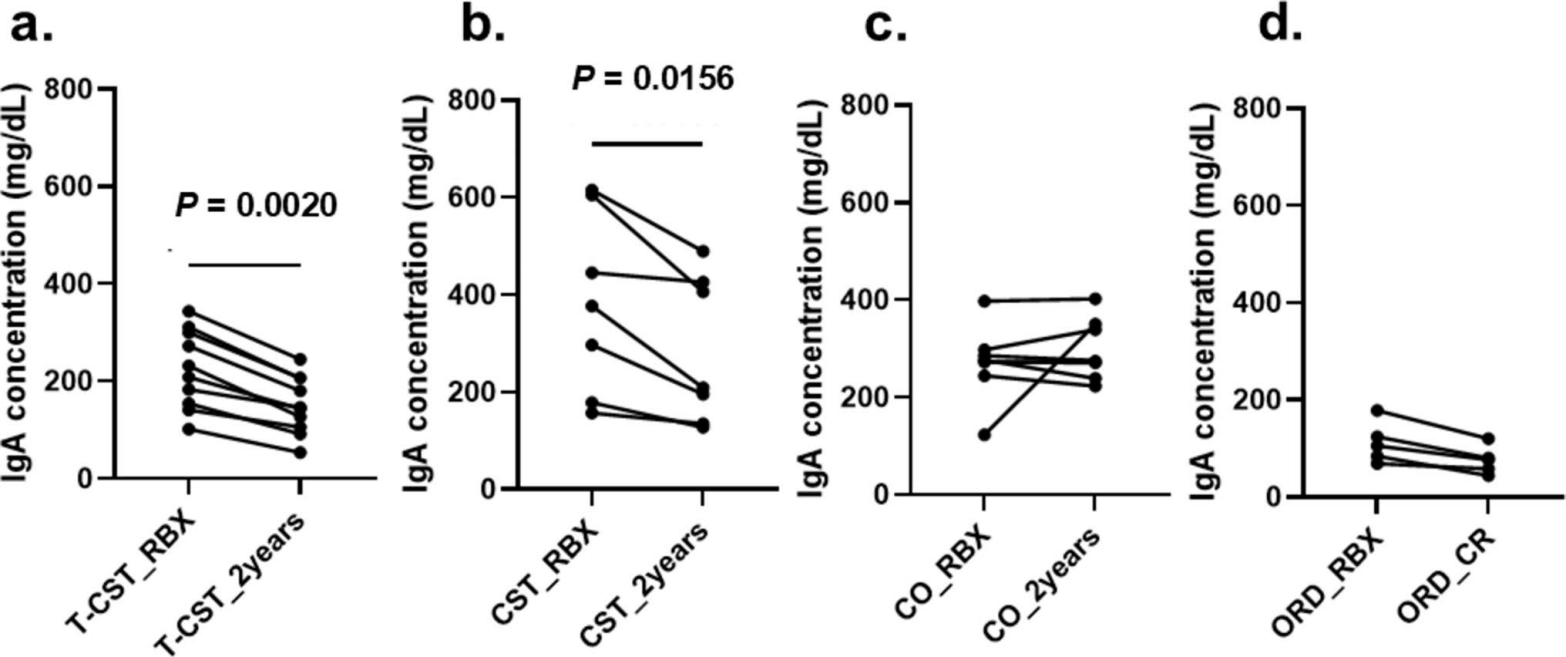
Changes in the serum IgA levels. **a** T-CST group, **b** CST group, **c** CO group, **d** ORD group. Serum IgA was quantified pre- and post-treatment. Serum IgA level significantly decreased in the T-CST (224.6 ± 80.65, 150.5 ± 59.15, *P* = 0.0020) and CST groups (382.2 ± 186.1, 284.3 ± 151.4, *P* = 0.0156) but not in the CO (271.3 ± 75.2, 296.6 ± 61.1, *P* = 0.9453) and ORD groups (112.4 ± 42.3, 75.97 ± 28.71, *P* = 0.0625). *P* value was calculated with the Wilcoxon matched-pairs signed-rank test. *T-CST* tonsillectomy and corticosteroid therapy, *CST* corticosteroid therapy, *CO* conservative therapy, *ORD* other renal diseases, *RBX* renal biopsy, *CR* complete remission

We quantified serum IgA using ELISA and analyzed whether the levels changed post-treatment compared with those at diagnosis in the T-CST, CST, CO, and ORD groups. The serum IgA levels significantly decreased post-treatment in the T-CST (224.6 ± 80.65, 150.5 ± 59.15, *P* = 0.0020) and CST groups (382.2 ± 186.1, 284.3 ± 151.4, *P* = 0.0156) but not in the CO (271.3 ± 75.2, 296.6 ± 61.1, *P* = 0.9453) and ORD groups (112.4 ± 42.3, 75.97 ± 28.71, *P* = 0.0625) (Fig. 3a–d).

### RA of IgA1 *O*-glycoforms in serum samples pre- and post-treatment

**Fig. 4:**
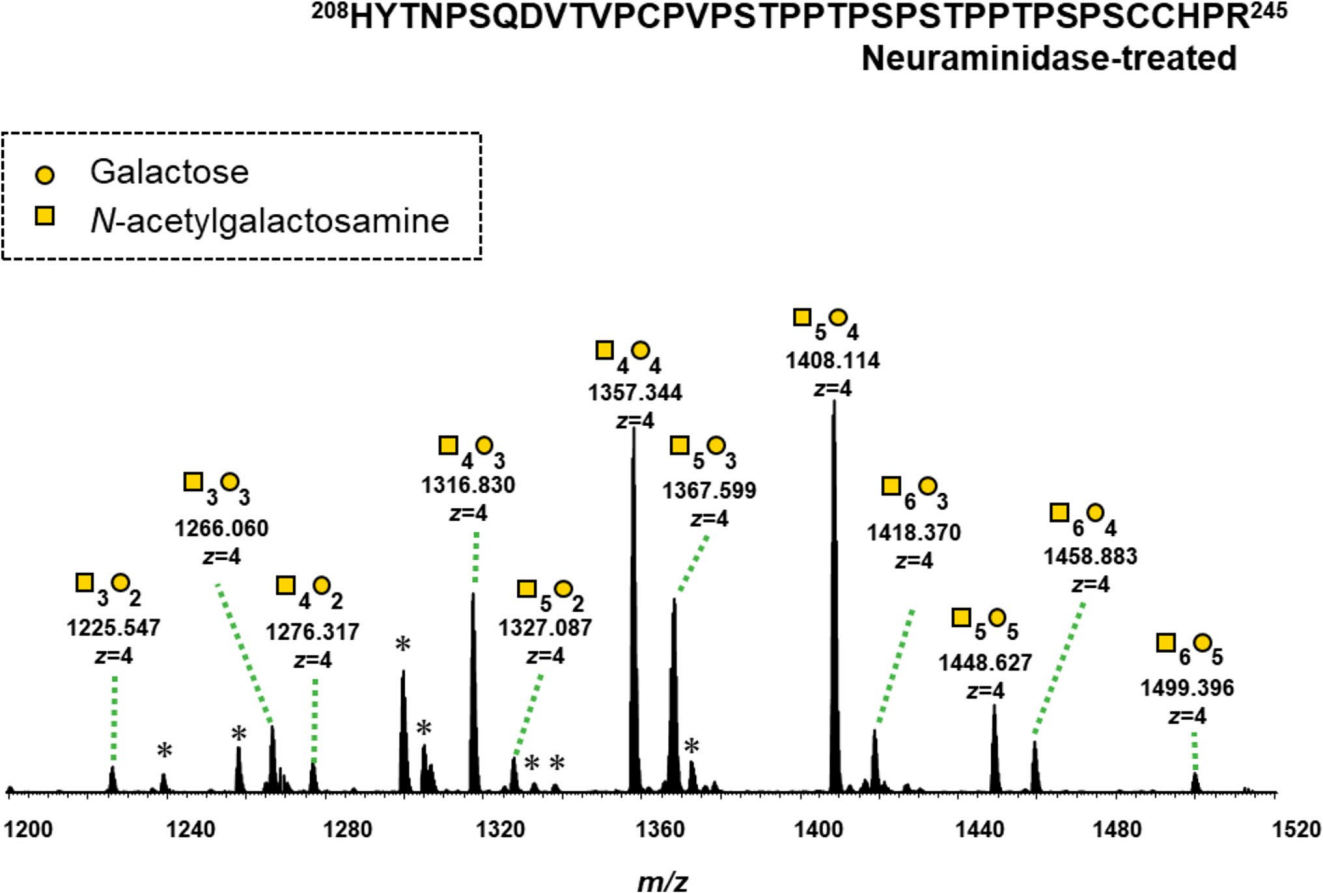
Representative mass spectrum of the desialylated tryptic fragments of IgA1 HR *O*-glycoforms. Twelve glycoforms were detected in the mass spectra at a retention time of 52–60 min. *Unassigned peaks with triply charged ions and not glycopeptides derived from IgA1 HR

Twelve glycoforms of desialylated IgA1 HR *O*-glycopeptides were detected in the mass spectra at retention time (RT) 52–60 min (Fig. 4). The *O*-glycoforms of IgA1 HR in the T-CST group showed significant changes in RA post-treatment compared with those in the CST, CO, and ORD groups. In the T-CST group, 7 of the 12 *O*-glycans showed significant differences in terms of RA post-treatment. Among them, the RA of 3GalNAc3Gal, which is the characteristic *O*-glycan phenotype in IgAN [13], tended to increase in the IgAN group compared with that in the ORD group, and it decreased significantly post-treatment only in T-CST group (*P* = 0.0195) (Table 2).

**Table 2:** Changes in the RA of IgA1 *O*-glycoforms pre- and post-treatment

*O*-glycans	GalNAc	Gal	Mass accuracy (ppm error)	IgAN (T-CST)		IgAN (CST)		IgAN (CO)		IgAN (RBX, total)	ORD		T-CST	CO	CST	ORD	IgAN vs. ORD
				RA, % (SD) (RBX) (*n* = 10)	RA, % (SD) (2 years) (*n* = 10)	RA, % (SD) (RBX) (*n* = 7)	RA, % (SD) (2 years) (*n* = 7)	RA, % (SD) (RBX) (*n* = 8)	RA, % (SD) (2 years) (*n* = 8)	RA, % (SD) (*n* = 25)	RA, % (SD) (RBX) (*n* = 5)	RA, % (SD) (CR) (*n* = 5)	Wilcoxon signed-rank test RBX vs. 2 years * P*-value	Wilcoxon signed-rank test RBX vs. 2 years * P*-value	Wilcoxon signed-rank test RBX vs. 2 years * P*-value	Wilcoxon signed-rank test RBX vs. CR *P*-value	Mann Whitney test *P*-value
3 Glycans	3	2	– 0.675	1.63 (0.69)	1.57 (0.73)	1.34 (1.05)	1.53 (0.33)	1.76 (0.39)	1.61 (0.34)	1.59 (0.73)	1.55 (0.27)	1.36 (0.30)	0.9805	0.0234*	0.9375	0.6250	0.8398
	3	3	– 0.613	4.78 (1.15)	4.44 (0.93)	5.89 (1.21)	5.75 (1.48)	5.57 (1.43)	5.31 (1.39)	5.34 (1.30)	4.17 (0.48)	3.99 (0.76)	0.0195*	0.1016	> 0.9999	0.4375	0.0507
4 Glycans	4	2	– 0.217	1.40 (0.75)	1.70 (0.79)	1.26 (0.85)	0.79 (0.86)	1.36 (0.41)	1.29 (0.35)	1.35 (0.67)	1.46 (0.27)	1.33 (0.19)	0.0137*	0.2344	0.1563	0.4375	0.2131
	4	3	– 0.476	10.05 (2.55)	10.97 (2.32)	10.06 (2.43)	9.54 (1.15)	10.87 (1.05)	10.33 (0.99)	10.32 (2.09)	10.54 (1.08)	9.59 (0.59)	0.0645	0.0078**	0.9375	0.1875	0.5971
	4	4	0.017	27.13 (2.78)	25.26 (2.04)	27.09 (2.09)	26.23 (2.53)	27.79 (2.71)	27.96 (2.54)	27.33 (2.50)	27.43 (1.69)	27.74 (2.99)	0.0039**	0.7422	0.1094	> 0.9999	0.8293
5 Glycans	5	2	– 0.736	1.20 (0.60)	1.48 (0.72)	0.82 (0.89)	1.19 (0.61)	1.15 (0.49)	1.17 (0.41)	1.08 (0.66)	1.29 (0.27)	1.23 (0.20)	0.0332*	0.8125	0.1563	0.6250	0.3204
	5	3	– 0.934	9.21 (2.03)	10.27 (2.01)	8.59 (1.61)	8.98 (2.47)	9.07 (1.37)	9.44 (1.63)	8.99 (1.68)	9.94 (0.94)	10.10 (0.93)	0.0020**	0.5469	0.4688	0.8125	0.2048
	5	4	0.194	27.62 (2.72)	27.59 (2.35)	27.57 (2.40)	27.92 (2.29)	26.60 (1.29)	26.91 (1.42)	27.28 (2.22)	27.63 (1.58)	28.42 (1.50)	0.9219	0.3125	0.6875	0.4375	0.7362
	5	5	– 0.053	8.07 (2.19)	7.41 (1.83)	9.34 (1.87)	9.89 (1.22)	7.68 (0.84)	7.86 (1.16)	8.33 (1.83)	7.53 (1.08)	7.67 (0.79)	0.0391*	0.6406	0.3750	0.6250	0.4812
6 Glycans	6	3	– 0.759	2.84 (0.46)	3.23 (0.46)	2.57 (0.49)	2.71 (0.40)	2.80 (0.66)	2.76 (0.52)	2.75 (0.53)	3.04 (0.89)	3.14 (1.16)	0.0039**	0.7813	0.1563	0.8750	0.5803
	6	4	– 0.498	3.48 (0.82)	3.54 (0.79)	3.37 (0.68)	3.43 (0.77)	3.17 (0.47)	3.11 (0.43)	3.35 (0.67)	3.25 (0.61)	3.21 (0.92)	0.4473	0.1484	0.9375	0.7500	0.9206
	6	5	– 0.418	1.74 (0.61)	1.63 (0.18)	1.63 (0.49)	1.60 (0.46)	1.46 (0.24)	1.42 (0.24)	1.62 (0.48)	1.39 (0.23)	1.36 (0.26)	0.2031	0.3828	0.9375	0.8125	0.2319

Data are presented as mean (standard deviation, SD). In the T-CST group, 7 of the 12 *O*-glycans showed significant differences in terms of RA post-treatment. Among them, 3GalNAc3Gal, which we previously found to be the characteristic *O*-glycan phenotype in IgAN, showed a tendency to increase in the IgAN group compared with that in the ORD group, and it decreased significantly post-treatment only in T-CST group. There was no change in the RA of *O*-glycoform in the CST and ORD group. *GalNAc* *N*-acetylgalactosamine, *Gal* galactose, *RA* relative abundance, *T-CST* tonsillectomy and corticosteroid therapy, *CST* corticosteroid therapy, *CO* conservative therapy, *ORD* other renal diseases, *RBX* renal biopsy, *CR* complete remission, * 0.01 ≤ *P* < 0.05, ** 0.001 ≤ *P* < 0.01, *** *P* < 0.001

**Fig. 5:**
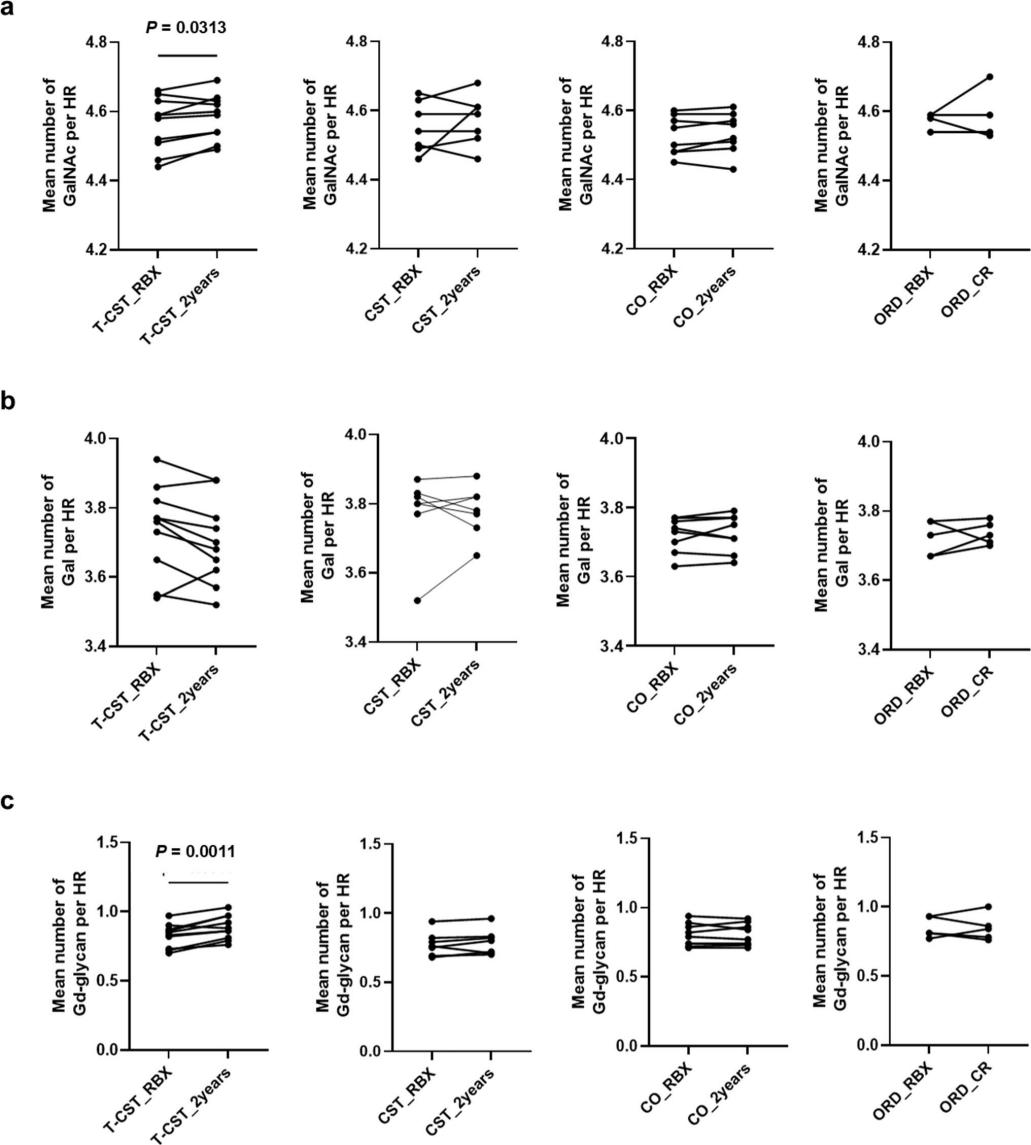
Changes in the content of GalNAc, Gal, and Gd-glycan in IgA1 HR. The mean number of GalNAc residues and Gd-glycans in the IgA1 HR significantly increased post-treatment only in the T-CST group (*P* = 0.0313 and* P* = 0.0011, respectively), but not in the CST, CO, and ORD groups. The mean number of Gal residues in the IgA1 HR remained unchanged in all groups. *GalNAc* *N*-acetylgalactosamine, *Gal* galactose, *Gd-glycan* galactose-deficient glycan, *HR* hinge region, *T-CST* tonsillectomy and corticosteroid therapy, *CO* conservative therapy, *ORD* other renal diseases, *RBX* renal biopsy, *CR* complete remission

The mean number of GalNAc residues and Gd-glycans in the IgA1 HR significantly increased only in the T-CST group from diagnosis to post-treatment (*P* = 0.0313, 0.0011), but not in the CST, CO, and ORD groups. The mean number of Gal residues per HR of IgA1 remained unchanged in all groups (Fig. 5).

### RA of IgA1 *N*-glycoforms between pre- and post-treatment

For IgA1 glycopeptides with Asn^340^, two different peptide sequences have been detected in a previous study using *N*-glycosidase F-treated samples [20]. We identified glycopeptides with LAGKPTHVNVSVVMAEVDGTCY at a RT of 68–73 min and glycopeptides with truncated LAGKPTHVNVSVVMAEVDGTC at a RT of 63–68 min. For IgA1 glycopeptides with Asn^144^, glycopeptides with LSLHRPALEDLLLGSEANLTCTLTGLR were identified at a RT of 87–92 min.

**Fig. 6:**
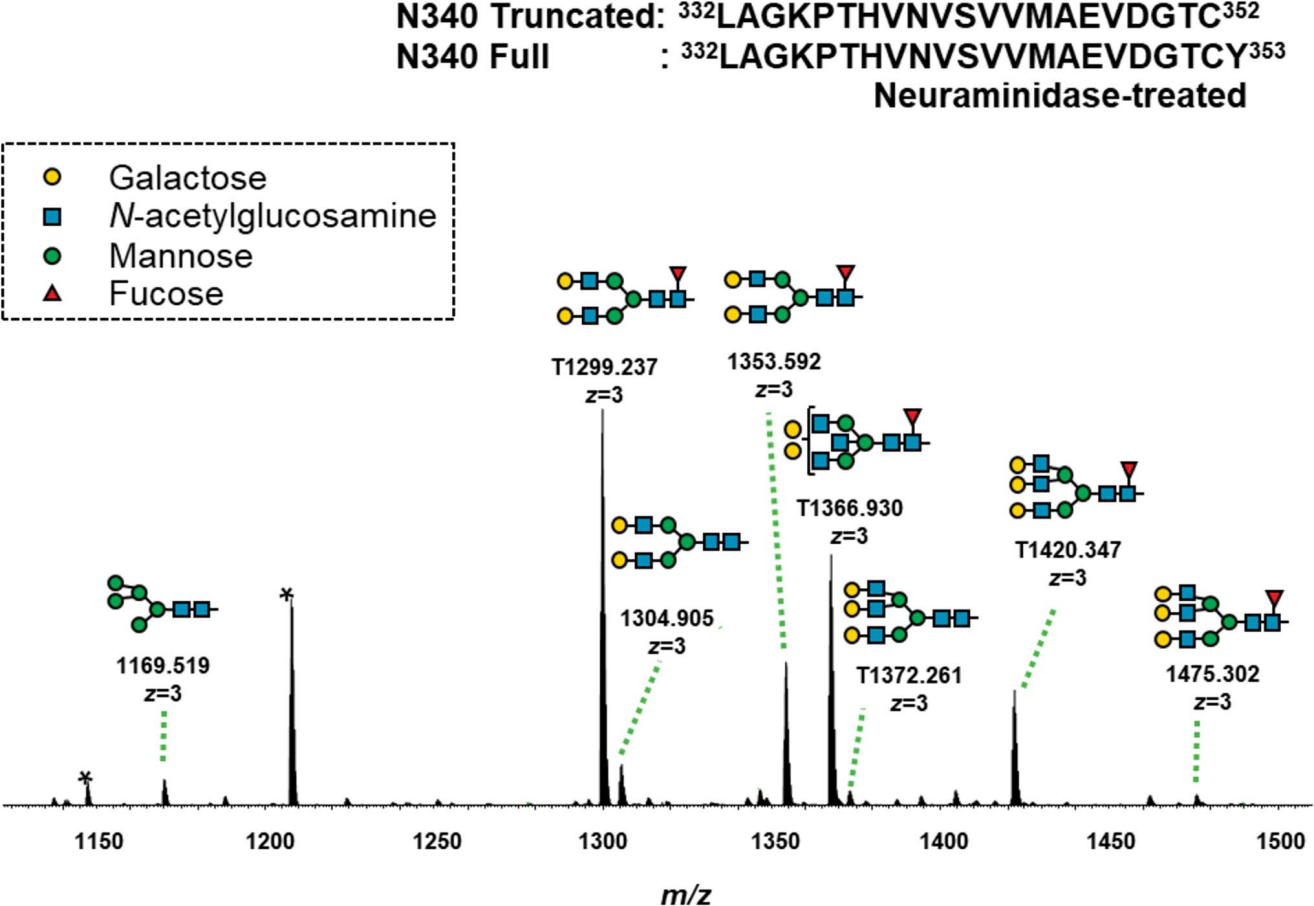
Representative mass spectrum of the desialylated tryptic fragments of IgA1 Fc *N*-glycopeptides at Asn^340^. The monoisotopic *m/z* value and charge number of the Fc* N*-glycopeptide ions are shown above individual peaks. “T” represents C-terminally truncated glycopeptides. * Represents peak other than the *N*-glycopeptide peak

**Fig. 7:**
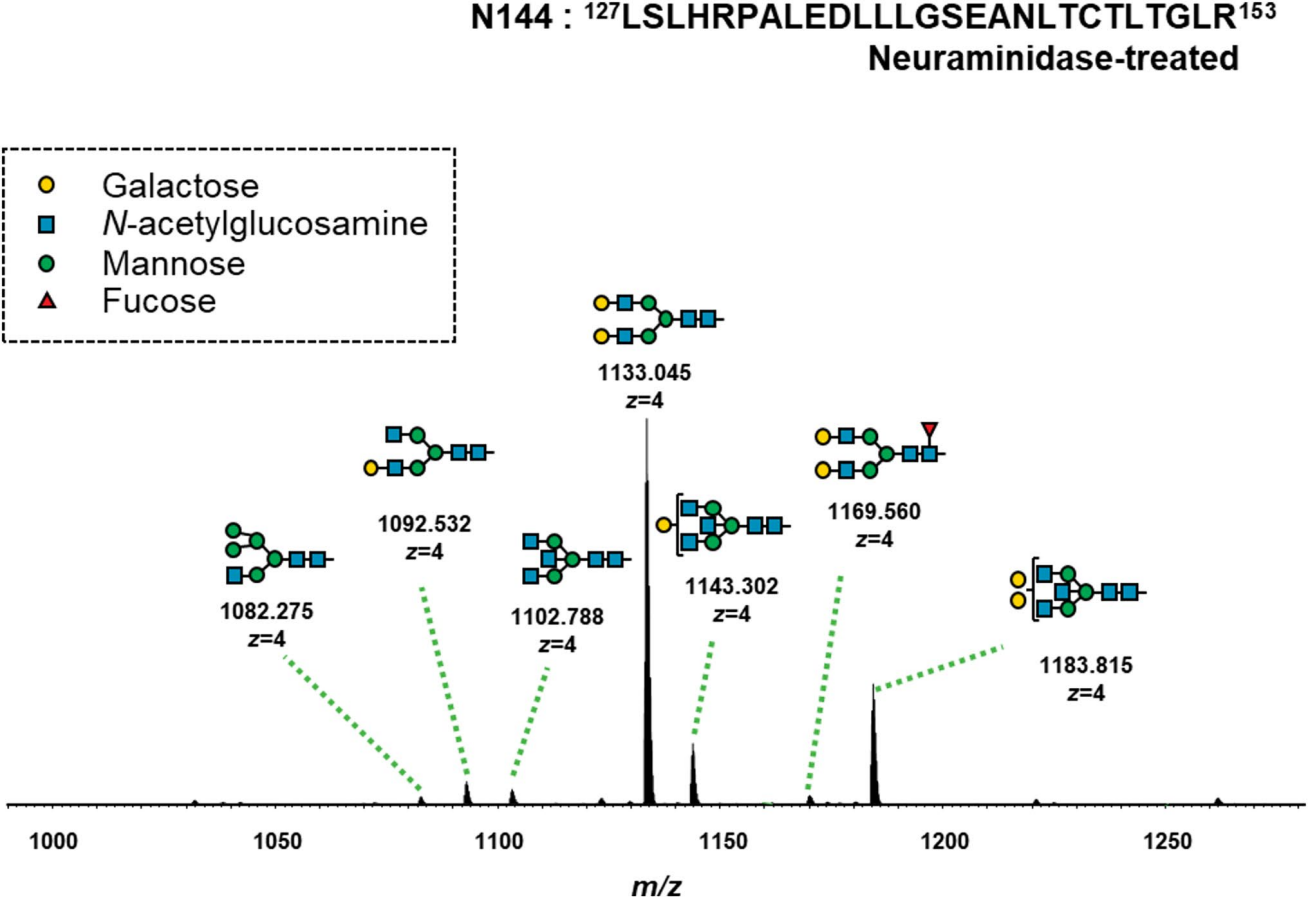
Representative mass spectrum of the desialylated tryptic fragments of IgA1 Fc *N*-glycopeptides at Asn^144^**.** The monoisotopic *m/z* value and charge number of the Fc* N*-glycopeptides ions are shown above the individual peaks

Six and seven *N*-glycopeptides in the IgA1 Fc region were detected at Asn^340^ and Asn^144^, respectively (Figs. 6 and 7, Tables 3 and 4). The biosynthesis of *N*-glycans proceeds from the initial attachment of precursor glycan through the processing and trimming of oligomannose structures, adding branching (in IgA1, usually biantennary, triantennary, and bisected glycans), elongation of branches, and core fucosylation; Table 5 shows glycan structures and MS profiles according to the *N*-glycan formation process. For Asn^340^, more than 90% of *N*-glycopeptides contained fucosylated complex glycans, as reported previously [4], and there were 0.5–4.9% glycopeptides with oligomannose type *N*-glycan (Tables 3 and 5). Furthermore, *N*-glycans at Asn^340^ were galactose-rich, that is, GlcNAc capped by Gal at high frequency (Table 5). Conversely, at Asn^144^, the RA of glycopeptides with fucosylated *N-*glycan was only 0.0%–1.8%, and glycopeptides with oligomannose type *N*-glycan were not detected (Tables 4 and 5). When comparing the *N*-glycans at Asn^340^ and Asn^144^ pre- and post-treatment, several differences were observed, mainly in the T-CST group. Among these, only fucosylation of *N*-glycan at Asn^340^ significantly increased in the IgAN group compared with that in the ORD group (*P* = 0.0189), and it decreased significantly post-treatment only in T-CST group (*P* = 0.0195) (Table 5).

**Table 3:**
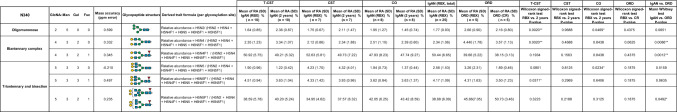
Changes in the RA of IgA1 *N*-glycoforms at Asn^340^ pre- and post-treatment

Six *N*-glycopeptides in the IgA1 Fc region were detected at Asn^340^. *GlcNAc* *N*-acetylglucosamine, *Man* mannose, *Gal* galactose, *Fuc* fucose, *H* hexose, *N* *N*-acetylhexosamine, *RA* relative abundance, *T-CST* tonsillectomy and corticosteroid therapy, *CST* corticosteroid therapy, *CO* conservative therapy, *ORD* other renal diseases, *RBX* renal biopsy, *CR* complete remission, * 0.01 ≤ *P* < 0.05, ** 0.001 ≤ *P* < 0.01

**Table 4:**
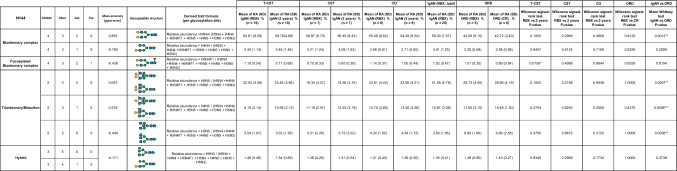
Changes in the RA of IgA1 *N*-glycoforms at Asn^144^ pre- and post-treatment

Seven *N*-glycopeptides in the IgA1 Fc region were detected at Asn^144^. *GlcNAc* *N*-acetylglucosamine, *Man* mannose, *Gal* galactose, *Fuc* fucose, *H* hexose, *N* *N*-acetylhexosamine, *RA* relative abundance, *T-CST* tonsillectomy and corticosteroid therapy, *CST* corticosteroid therapy, *CO* conservative therapy, *ORD* other renal diseases, *RBX* renal biopsy, *CR* complete remission, * 0.01 ≤ *P* < 0.05, ** 0.001 ≤ *P* < 0.01

**Table 5:**
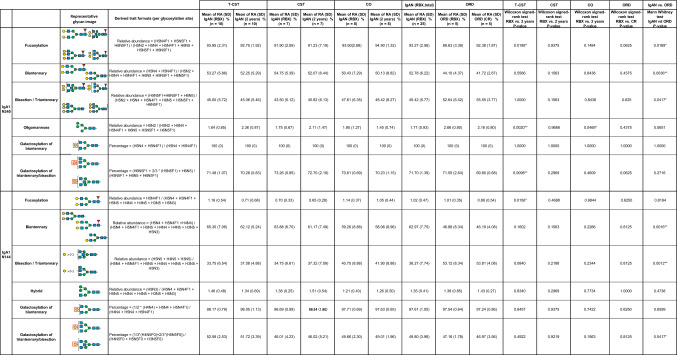
Derived IgA glycosylation traits as determined from detected glycopeptides

Glycan structures and MS profiles according to the *N*-glyacan formation process are shown in this table. The RA of fucosylated (% of fucosylated *N*-glycopeptides), biantennary (% of *N*-glycopeptides carrying a biantennary *N*-glycan), bisecting (% of *N*-glycopeptides carrying a bisecting GlcNAc), triantennary (% of *N*-glycopeptides carrying a triantennary *N*-glycan), hybrid (% of *N*-glycopeptides carrying a hybrid *N*-glycan), and oligomannose (% of *N*-glycopeptides carrying an oligomannose *N*-glycan) *N*-glycopeptides at Asn^340^ and Asn^144^ were calculated using the formulae provided in the table. For Asn^340^, more than 90% of *N*-glycopeptides contained fucosylated complex glycans, and there were 0.5–4.9% glycopeptides with oligomannose type *N*-glycan. Conversely, at Asn^144^, the RA of glycopeptides with fucosylated *N*-glycan was only 0.0–1.8%, and glycopeptides with oligomannose type *N*-glycan were not detected. When comparing the general frame work of *N*-glycoforms at Asn^340^ and Asn^144^ pre- and post-treatment, several differences were observed, mainly in the T-CST group. Among them, only fucosylation of *N*-glycan at Asn^340^ significantly increased in the IgAN group compared with that in the ORD group (*P* = 0.0189), and it decreased significantly post-treatment only in the T-CST group (*P* = 0.0195). *GlcNAc* *N*-acetylglucosamine, *Man* mannose, *Gal* galactose, *Fuc* fucose, *H* hexose, *N* *N*-acetylhexosamine, *RA* relative abundance, *T-CST* tonsillectomy and corticosteroid therapy, *CST* corticosteroid therapy, *CO* conservative therapy, *ORD* other renal diseases, *RBX* renal biopsy, *CR* complete remission, * 0.01 ≤ *P* < 0.05, ** 0.001 ≤ *P* < 0.01

## Discussion

Recently, a relationship between changes in both *O*- and *N*-glycosylation of IgA1 and IgAN was demonstrated using HRMS [12–15]. In this study, we investigated longitudinal changes in the RA of IgA1* O*- and *N*-glycoforms in patients with IgAN and other renal diseases using the workflow that we developed [5].

We demonstrated that the number of GalNAc residues per HR increased after treatment and that the RA of IgA1 with 3GalNAc3Gal decreased after treatment in the T-CST group but not in the CST, CO, and ORD groups. We also determined a difference in the *N*-glycosylation of IgA1 pre- and post-treatment in the T-CST group. The RA of fucosylated *N*-glycan at Asn^340^ increased in the IgAN group compared with that in the ORD group, and it decreased post-treatment in the T-CST group.

As for *O*-glycosylation, a reduced number of GalNAc residues in IgA1 HR is a common characteristic of patients with IgAN across racial groups and increased RA of IgA1 with 3GalNAc3Gal is a characteristic of IgAN in Asians, as we reported previously [13, 14]. Furthermore, a reduced number of GalNAc residues in IgA1 HR is characteristic of patients with crescentic IgAN [15]. The finding that IgA1 with reduced number of *O*-glycans decreased post-treatment in the T-CST group is in agreement with the involvement of these glycoforms in the pathogenesis of IgAN.

Although not much is known about the association of IgA1 *N*-glycans with IgAN, changes in the* N*-glycans of IgG are well documented in some chronic inflammatory and autoimmune diseases. For example, fucosylation of anti-dsDNA IgG1 has been reported to correlate with disease activity in patients with treatment-naïve systemic lupus erythematosus [21, 22]. It was also shown that removal of the core fucosylation leads to increased binding affinity to the Fcγ receptor Шa due to increased carbohydrate–carbohydrate interactions with the *N*-glycans of the Fcγ receptor promoting substantially increased antibody-dependent cellular cytotoxicity (ADCC) [23–25]. Despite the importance of IgG glycosylation, available data about the interaction of IgA with the Fcα receptor (FcαRI), also known as CD89, do not clarify whether distinct IgA glycans play a role in the receptor interaction [26–28]. To our knowledge, this is the first study to show that fucosylation of *N*-glycan at Asn^340^ increased in the IgAN group compared with that in the ORD group at diagnosis and that it decreased post-treatment in the T-CST group. It is still unclear what the role of elevated fucosylation of IgA1 *N-*glycan is in biology of IgA1, warranting further investigation.

The mechanisms involved in altered representation of IgA1 *O*-glycoforms and *N*-glycoforms were not examined in the present study. Palatine tonsillectomy may have affected the changes in IgA1 glycosylation. Recently, the association between IgAN and abnormalities in the mucosal lymphoid-associated tissue (MALT) has been gaining attention [29]. It has been postulated that patients with IgAN have IgA1-producing cells with altered homing receptors when migrating from an inductive site to the effector site and therefore, they “home” in the bone marrow by “mistake” [30, 31]. This process ultimately results in elevated amounts of circulated mucosal-type IgA1, i.e., polymeric IgA1 with distinct glycosylation. In this study, we determined that the serum IgA levels at diagnosis were significantly higher in the IgAN group than in the ORD group. Serum IgA levels significantly decreased post-treatment compared to their pre-treatment levels in the T-CST and CST group. However, high serum IgA levels were observed post-treatment in some patients in the CST group. Furthermore, the abundance of *O*- and *N*-glycoforms of IgA1 changed post T-CST treatment in this group but remained unchanged in the CST, CO, and ORD groups. Thus, palatine tonsillectomy may have contributed to the decrease in MALT-derived IgA1-secreting B cells and, consequently in altered levels and glycosylation of circulatory IgA1. It would be interesting to understand which changes in IgA1 glycoforms can predict whether tonsillectomy is effective or not, but this was not evident in this study.

Another possible explanation for variation of IgA1 glycoforms could be the role of cytokines in altered expression of specific *O*-glycosyltransferases. For example, IL-6 can reduce the expression of core 1 beta1,3-galactosyl-transferase (C1GalT1 protein encoded by *C1GALT1* gene) and C1GalT1-specific chaperone (encoded by *C1GALT1C1* gene); the C1GalT1 enzyme adds galactose to GalNAc residues of *O*-glycans. IL-6 can also increase the expression of α-*N*-acetylgalactosaminide α-2,6-sialyltransferase 2 (encoded by *ST6GALNAC2* gene) in B cells of patients with IgAN that may lead to premature sialylation of GalNAc that blocks normal galactosylation [32]. Furthermore, human naïve B cells co-stimulated with CD40L and IL-21 in vitro reportedly showed reduced expression of polypeptide GalNAc-transferase 2 (ppGalNAc-T2) compared with cell stimulated with CD40L alone or with CD40L and IL-10 [33]. Although there have been no reports on the relationship between IgA1 *N*-glycosylation and cytokines, a relationship between cytokines and IgG *N*-glycosylation has been reported [34, 35]. Palatine tonsillectomy and corticosteroid therapy may contribute to a decrease in proinflammatory cytokine levels, which are elevated in patients with IgAN, and may induce changes in the *O*- and *N*-glycosylation of IgA1.

This study has two limitations. First, it was not possible to evaluate the efficacy of tonsillectomy based on changes in the abundance of IgA1 *O*- and *N*-glycoforms. Further studies are needed to identify potential of specific glycoforms as biomarkers predictive of the efficacy of tonsillectomy. Second, we could not clarify how altered abundance of IgA1 glycoforms in serum from pre- and post-treatment implicates these glycoforms in the pathogenesis of IgAN and whether the altered representation of different glycoforms may be associated with disease exacerbation or protection. Further investigation is needed to clarify how IgA1 glycoforms with these glycan structures are involved in IgAN pathogenesis.

## Conclusion

We demonstrated that the MS analysis of *O*- and *N*-glycoforms of IgA1 revealed substantial changes in their abundance in the patients with IgAN who underwent both tonsillectomy and corticosteroid therapy. These findings provide new insights into the development of novel glycobiomarkers for monitoring treatment responses, with implications for risk assessment of IgAN recurrence.

## Supplementary Information

Below is the link to the electronic supplementary material.

Supplementary file1 (DOCX 227 KB)

Supplementary file2 (XLSX 62 KB)

## Data Availability

All data supporting the findings of this study are included in Supplementary Table S1 Any requests for additional details are welcome and can be directed towards the corresponding authors.
