# Supplementary material for: Longitudinal changes in the abundance of IgA1 O- and N-glycoforms in IgA nephropathy
Source: Clin Exp Nephrol. 2025 Apr 7;29(8):1102–15. doi: 10.1007/s10157-025-02659-y (PMC12331428; doi:10.1007/s10157-025-02659-y)
Supplement: Supplementary file 1 — Supplementary file1 (DOCX 227 KB) [file 10157_2025_2659_MOESM1_ESM.docx]

**Longitudinal changes in the abundance of IgA1 *O-* and *N*-glycoforms in IgA nephropathy**

***Clinical and Experimental Nephrology***

*Supplementary Information*

**Masaya Hirayama^1,2^, Yukako Ohyama^1,3^, Yudai Tsuji^1^, Tetsuro Enomoto^4^, Midori Hasegawa^3^, Naotake Tsuboi^3^, Jan Novak^5^, Kazuo Takahashi^1,3^**

**^1^Department of Biomedical Molecular Sciences, Fujita Health University School of Medicine, 1-98 Dengakugakubo, Kutsukake-cho, Toyoake, Aichi 470-1192, Japan.**

**^2^Department of Pathology and Cytopathology, Fujita Health University School of Medical Sciences, 1-98 Dengakugakubo, Kutsukake-cho, Toyoake, Aichi 470-1192, Japan.**

**^3^Department of Nephrology, Fujita Health University School of Medicine, 1-98 Dengakugakubo, Kutsukake-cho, Toyoake, Aichi 470-1192, Japan.**

**^4^Oriental Yeast Co., Ltd., 50 Kanou-cho, Nagahama, Shiga 526-0804, Japan.**

**^5^Department of Microbiology, University of Alabama at Birmingham, 1720 2^nd^ Ave South, Birmingham, AL 35294, USA**

**^*^Correspondence to:**

**E-mail: kazuot@fujita-hu.ac.jp (K. Takahashi)**

**Tel: +81-562-93-2430 (Office), -2431 (Lab), -9245 (Nephrology)**

**Fax: +81-562-93-1830**

**Supplementary Table and Figure Legends**

**Supplementary Figure S1.** Flowchart of the selection of treatment options.

IgAN, IgA nephropathy; eGFR, estimated glomerular filtration rate; CKD, chronic kidney disease; UP, urinary protein; RASI, renin-angiotensin-system inhibitors; RBC, red blood cell; WBC, white blood cell.


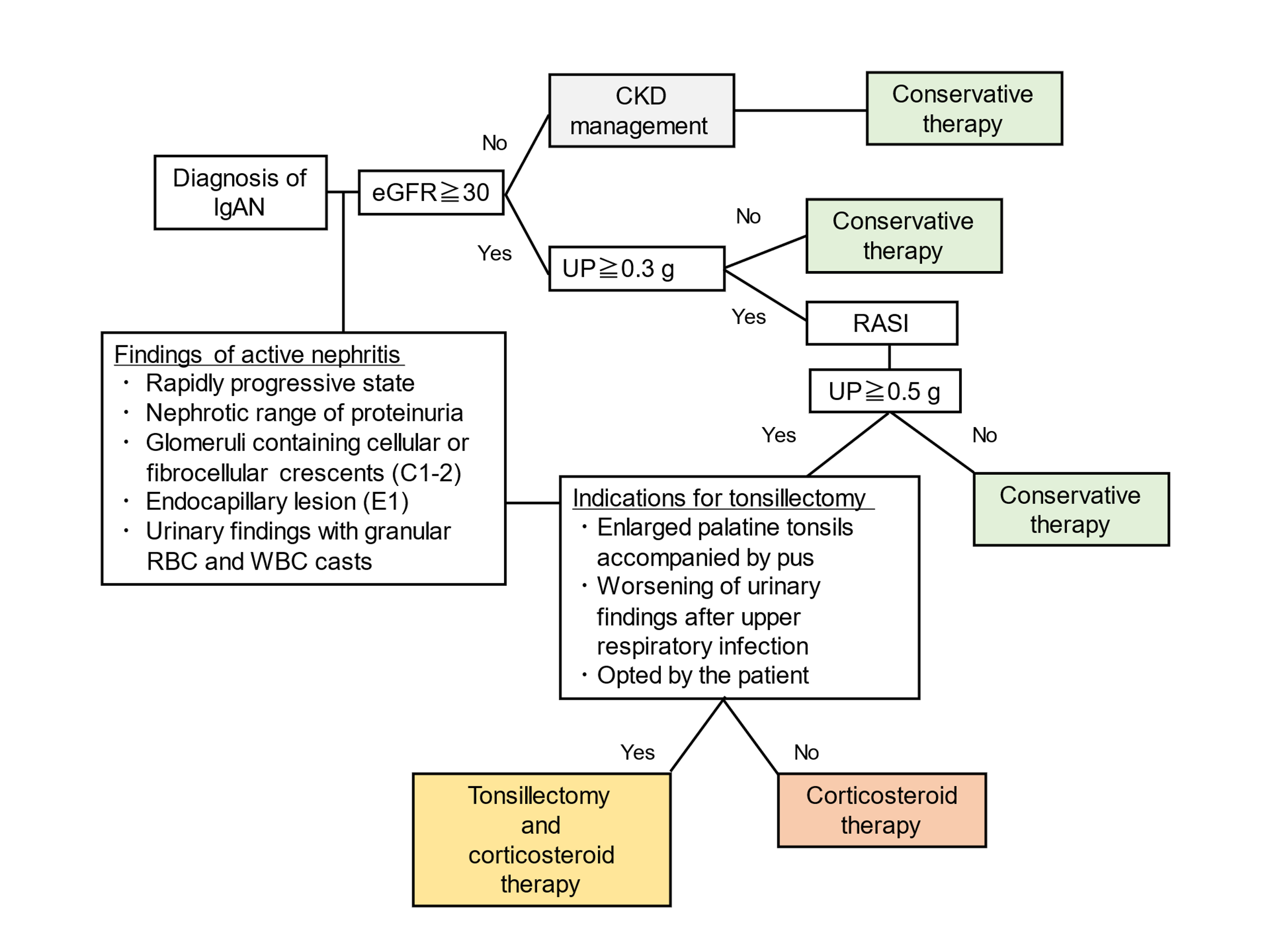


**Supplementary Table S1.**

**Supplementary Table S1.** The data set supporting the findings of this study.

Gd-IgA1, galactose-deficient IgA1; MAP, mean arterial pressure; Cr, serum creatinine concentration; eGFR, estimated glomerular filtration rate; HPF, high power field; RA, relative abundance; GalNAc, *N*-acetylglucosamine; Gal, galactose; Gd-glycan, galactose-deficient glycan; HR, hinge region; H, hexose; N, *N*-acetylhexosamine; F, fucose; M, mesangial proliferation; E, endocapillary proliferation; S, segmental glomerulosclerosis; T, interstitial fibrosis/tubular atrophy; C, crescents.
